# Key factors for implementing inhaler regimen switches in respiratory diseases: international expert consensus generated using a modified nominal group technique (NGT)

**DOI:** 10.1038/s41533-026-00489-3

**Published:** 2026-02-24

**Authors:** Omar S. Usmani, Nicolas Roche, Job F. M. van Boven, Jane Scullion, José Miguel Padilha, Tonya Winders, Andreja Šajnić, John P. Bell, Anna Lawson, Alison Evans, Heather Wellam, Adam Ben Taieb, Clare Foy, Noah Froud, Janwillem Kocks

**Affiliations:** 1 National Heart and Lung Institute, Imperial College London, London, UK; 2 Hôpital Cochin, Service de Pneumologie, Paris, France; 3 Department of Clinical Pharmacy and Pharmacology, Groningen Research Institute for Asthma and COPD (GRIAC), University Medical Center Groningen, University of Groningen, Groningen, The Netherlands; 4 University Hospitals of Leicester, Leicester, UK; 5 Nursing School of Porto, RISE-HEALTH, Porto, Portugal; 6 Global Allergy and Airways Patient Platform (GAAPP), Vienna, Austria; 7 University Hospital Centre Zagreb, Zagreb, Croatia; 8 Biopharmaceuticals Medical, AstraZeneca, Baar, Switzerland; 9 Global Corporate Affairs, AstraZeneca, Cambridge, UK; 10 Petauri Evidence, Bicester, UK; 11 Syneos Health Communications, London, UK; 12 General Practitioners Research Institute (GPRI), Groningen, The Netherlands; Observational and Pragmatic Research Institute, Singapore; Groningen Research Institute Asthma and COPD (GRIAC), University of Groningen, University Medical Center Groningen, Groningen, The Netherlands; Dept of Pulmonology, University of Groningen, University Medical Center Groningen, Groningen, The Netherlands

**Keywords:** Diseases, Health care, Medical research

## Abstract

To develop international expert consensus statements on when and how to implement inhaler switches for patients with asthma or chronic obstructive pulmonary disease, informing clinicians and decision-makers on appropriate circumstances, stakeholder roles, and essential steps for safe and effective switching. An international panel of eight clinical, payer, and patient experts participated in a nominal group technique. Ideas were generated in response to four research questions and rated on a 7-point Likert scale (1 = not at all important; 7 = extremely important). The decision-making unit framework was applied for stakeholder mapping. Clinical and patient-focused drivers were identified as the most important drivers for inhaler switching (rated very or extremely important by >60% of experts), which included inadequate disease control, inhaler technique errors, switching to maintenance and reliever therapy, adding a spacer, and addressing poor satisfaction. Operational factors such as supply shortages were considered moderately important, while environmental and cost-related drivers were considered least important (rated not at all important to neutral by >60% of experts). Inappropriate circumstances for inhaler switching centred on patient safety (rated very or extremely important by >85% of experts), including lack of consultation, consent, education, or follow-up, switching clinically stable patients, and introducing complex regimens. Of essential activities required for a consultation, experts estimated a median time of 36 min would be required. Experts suggest inhaler switches should prioritise clinical need and patient involvement over cost or sustainability goals, and suggest policymakers consider the time and complexity required for implementation at scale.

## Introduction

Globally, chronic respiratory diseases affect over 500 million people and are responsible for 4 million deaths annually^1^. They represent a significant clinical and economic burden^1^ for patients, carers and health systems^2–5^. Inhaled medications play a key role in the everyday management and control of common respiratory diseases^6^. In asthma, inhaled corticosteroids are the ‘cornerstone of care for asthma patients of all severity’^7^, treating underlying inflammation, and preventing symptoms, attacks and mortality^8^. In chronic obstructive pulmonary disease (COPD), inhaled medicines can improve symptom control and prevent exacerbations and mortality^9^.

The many device-drug combinations available promotes diversity of choice for clinicians and patients^10^. Device selection (e.g. pressurised metered dose inhalers, soft mist inhalers, dry powder inhalers, or nebulisers) can be as important as the active pharmaceutical ingredients to achieve optimal outcomes^11^. Numerous reasons may prompt consideration of substituting or switching inhaler regimens. Clinical and patient-focused factors that could drive such consideration include uncontrolled disease or exacerbations, side effects, low adherence or incorrect inhaler technique^11^. Regimen simplification, e.g. switching from multiple inhalers to a single device or prescribing the same inhaler type for concomitant medications, can improve adherence and outcomes, and may be a clinical reason for regimen switching^12^.

Non-clinical issues such as regulatory, economic^13,14^, environmental^15,16^, local guidelines, access, and supply may also drive inhaler switching. A recent Dutch survey showed that 60% of inhaler regimens were switched in the prior year and 60% of switches were for non-medical reasons^17^.

Experts note that switching an inhaler device solely for non-clinical reasons should be avoided, especially without clinical consultation^18,19^. Similarly, others have stated that patients with stable disease should preferably not have their medication changed^20^. Switching inhaler regimens without consultation can lead to decreased adherence and incorrect inhaler usage, contributing to poor disease control in COPD and asthma^21–23^. Reduced adherence also contributes to increased morbidity, mortality, and associated costs^24^. A systematic literature review of real-world evidence on the impact of switching inhaler regimens for non-clinical reasons showed that it can be detrimental to the patient-healthcare professional (HCP) relationship, especially for non-consented switches^25^. Critically, following any inhaler switch, patients must be assessed for inhaler errors, which are linked to worsened healthcare outcomes and an increased burden on healthcare resource utilisation (HCRU)^26^. Thus, switching inhaler regimens for non-clinical reasons is complex and can have highly variable clinical consequences, although high quality data on clinical outcomes are scarce^25^.

Previous guidance outlined how to optimise inhaler regimen switching, e.g. assessing patients’ control and inhalation abilities, and shared decision-making^11^. However, there is a lack of clear expert consensus on what constitutes appropriate and safe device switching and a robust framework is needed to inform best practice and guide personalised inhaler switching^25^. To address this, an international respiratory disease expert panel was convened; the panel developed a set of quality statements defining when inhaler regimen switching is/is not appropriate, which stakeholders should be involved, and the key steps and clinical time required to implement an appropriate switch. The intended audience for the results of the consensus is HCPs, policymakers, and wider decision-makers.

## Methods

### Study design

A nominal group technique (NGT) was used to develop expert consensus statements. NGT is a structured, facilitated approach that enables equal participation, systematic idea generation, and prioritisation through independent rating, making it suitable for informing policy and guideline development where empirical evidence is limited. This method combines individual input with group discussion and relies on anonymous scoring to reduce bias^27–29^. The process was conducted virtually and split into two sessions (January and February 2023) to accommodate participant availability. These were the only deviations from standard NGT methodology.

### Safeguards

The expert panel was convened by a pharmaceutical company sponsor; however, the sponsor’s role was limited to logistical support and attendance. The sponsor did not participate in discussions, influence content, or contribute to statement formulation. To ensure impartiality, an independent agency facilitated the meetings, acted as data custodian, and provided methodological oversight. These measures ensured transparency and independence of the consensus process.

**Protocol steps**

**Fig. 1:**
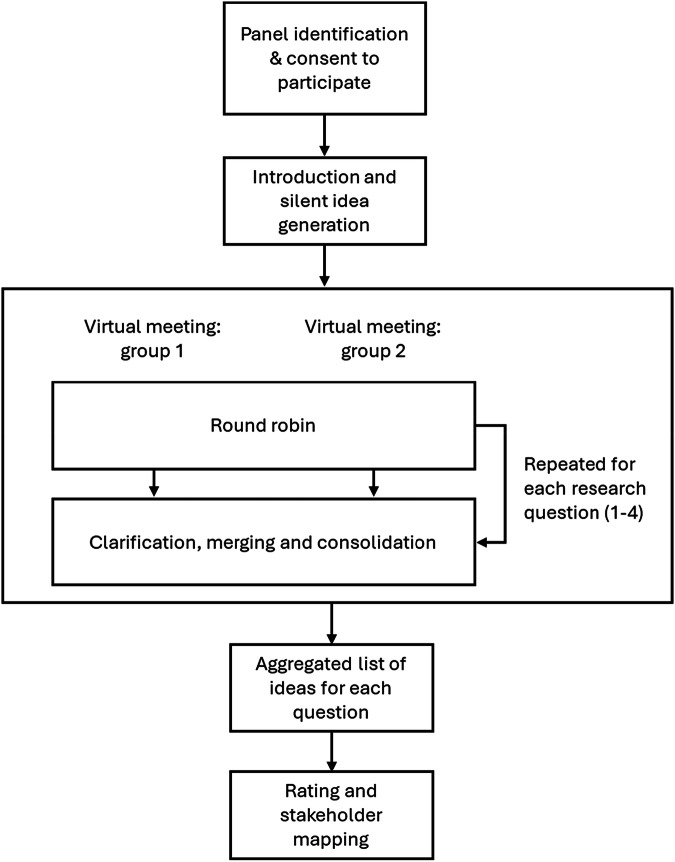
Illustration of the steps involved in this vNGT protocol.

The vNGT process comprised six stages (Fig. 1).

1. Panel identification and consent to participate
  Sixteen potential participants were identified through structured searches of professional directories, academic publications, and society memberships. Inclusion criteria focused on clinical experience in inhaled respiratory care and research, demonstrated through engagement with respiratory organisations, conference presentations, and published works. One patient advocate was included to represent the respiratory disease community; although not living with asthma or COPD, she holds a leadership role in the Allergy and Asthma Network and the Global Allergy and Airways Patient Platform (GAAPP), representing over 130 patient organisations worldwide. Eight participants were selected (recommended NGT size: 5–9). Identities were not anonymised during discussions. No reimbursement was provided. Declared independence and conflicts of interest are disclosed at the end of the manuscript.
2. Introduction and silent idea generation
  Participants received a recently published systematic review on non-clinical inhaler regimen switches prior to the meeting^25^. They were asked to consider patients with asthma or COPD who might undergo an inhaler regimen switch (device, drug, or both) and respond to four research questions:
  Q1: In what circumstances are inhaler regimen switches appropriate?
  Q2: In what circumstances are inhaler regimen switches not appropriate?
  Q3: What essential activities and timings are required for a successful inhaler regimen switch?
  Q4: Who should be involved in decision-making and implementation?
  Ideas were generated independently before the meeting.
3. Round robin
  During each virtual session, participants presented one idea at a time in a structured sequence, ensuring equal opportunity for contribution. The facilitator invited each participant in turn to share a single response to the research question, continuing the cycle until no new ideas emerged. This iterative approach prevented dominance by any individual and ensured comprehensive idea capture. All ideas were recorded in real time on a shared platform visible to participants, promoting transparency and enabling immediate clarification if needed. The facilitator’s role was limited to maintaining structure and neutrality, without influencing content.
4. Clarification, merging and consolidation
  During the facilitated discussion, overlapping or duplicate ideas were identified and merged collaboratively as part of the NGT process. This merging occurred either in real time during the meeting or subsequently using a secure, cloud-based platform designed for asynchronous engagement. This platform allowed both groups to review and clarify contributions from the other group, ensuring transparency and consensus on the final set of statements. The merging process was therefore integral to the structured NGT methodology and relied on expert judgment rather than post-hoc statistical analysis.
5. Rating
  Post meeting, participants rated statements anonymously via a secure, cloud-based platform. Items generated in response to research questions 1, 2 and 3 were scored for importance using a 7-point Likert scale (1 = not at all important; 7 = extremely important). For research question 3, estimated time (in min) was also collected for each activity.
6. Stakeholder mapping

For research question 4, roles were assigned using the Decision-Making Unit (DMU) framework, which defines six roles: initiator, gatekeeper, buyer, decider, influencer, and user^30^. Definitions were provided to ensure consistency, and experts could assign multiple roles per stakeholder.

- Initiator: identifies the need for an inhaler regimen switch.
- Gatekeeper: controls access to, or availability of, specific inhaler regimens.
- Buyer: provides funds for the inhaler regimen prescription or makes the purchase.
- Decider: takes the decision to make an inhaler regimen switch.
- Influencer: informs the inhaler regimen switch through advice and expertise.
- User: the individual who will use the new inhaler regimen.

Following this, experts listed core requirements for individuals conducting patient discussions during inhaler regimen switches.

### Ethics and data protection

Ethical approval was not required as the study involved expert consultation for service development rather than patient-level research. All participants provided informed consent prior to participation. Data were handled in compliance with applicable data protection regulations (e.g., GDPR), stored on secure, access-controlled platforms, and used solely for the purposes of consensus development. Anonymity was maintained during rating exercises, and no individual responses were disclosed. The full protocol, including data handling procedures, is available as supplemental material.

## Results

### Participants

The expert panel consisted of eight participants from six countries: Croatia, France, Portugal, The Netherlands, United Kingdom, and United States. Disciplines represented included nursing (n = 3), respiratory medicine (n = 2), general practice (n = 1), health economics/pharmacy (n = 1), and patient advocacy (n = 1). Table 1 provides an overview of the panel composition, including each participant’s professional title, area of specialty, country of residence, and declared independence.

**Table 1:** Expert panel title, specialist area, country and declared independence.

Expert ID	Title	Specialist area	Country	Declared independence
1	Professor of Respiratory Medicine	Asthma, COPD, chronic cough.	England	The research was conducted, interpreted, and reported impartially, free from undue influence or bias from the funding body.
2	Professor of Respiratory Medicine	COPD, asthma, inhaled therapy, real-world research.	France	The research was conducted, interpreted, and reported impartially, free from undue influence or bias from the funding body.
3	Associate Professor	Health economics, asthma, COPD, lung cancer.	Netherlands	The research was conducted, interpreted, and reported impartially, free from undue influence or bias from the funding body.
4	Consultant Respiratory Nurse	Respiratory conditions.	England	The research was conducted, interpreted, and reported impartially, free from undue influence or bias from the funding body.
5	Associate Professor of Nursing	COPD, nursing education.	Portugal	The research was conducted, interpreted, and reported impartially, free from undue influence or bias from the funding body.
6	President and CEO of Allergy & Asthma Network. President of the Global Allergy & Airways Patient Platform	Patient advocacy for asthma, allergy and related conditions.	USA	The research was conducted, interpreted, and reported impartially, free from undue influence or bias from the funding body.
7	Healthcare Manager; President of International Coalition of Respiratory nurses	Respiratory nursing.	Croatia	The research was conducted, interpreted, and reported impartially, free from undue influence or bias from the funding body.
8	General Practitioner; Professor of Inhalation Medicine; IPCRG Board Director	Asthma, COPD.	Netherlands	The research was conducted, interpreted, and reported impartially, free from undue influence or bias from the funding body.

### Consensus statements

**Fig. 2:**
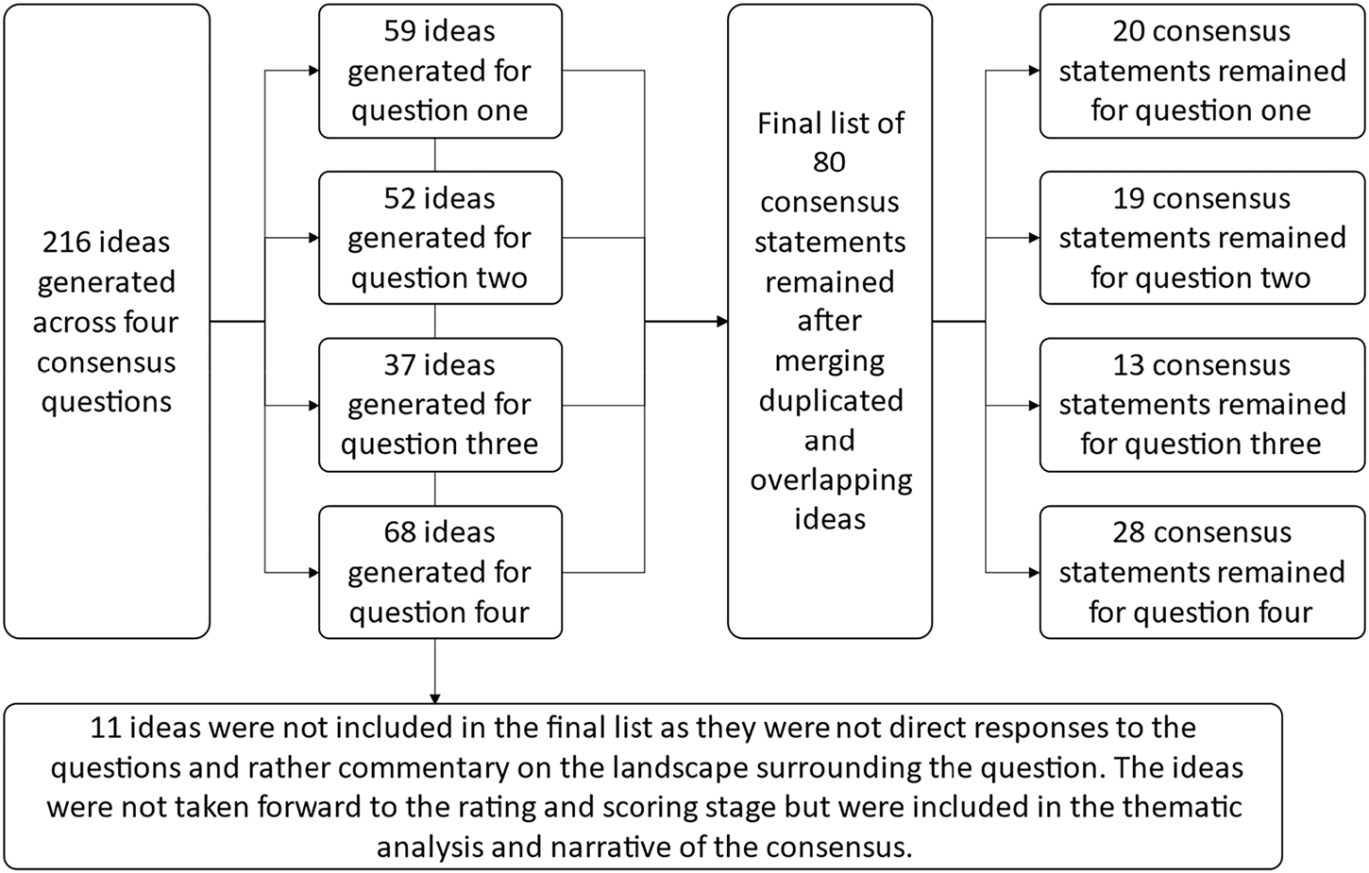
Number of ideas and consensus statements generated across questions. The four consensus ues ons were 1. In what circumstances are inhaler regimen switches appropriate. In what circumstances are inhaler regimen switches not appropriate 3. hat are the essen al ac vi es that need to be taken to implement a successful switch. In cases where inhaler regimen switches are appropriate, who should be involved in the decision making and implementa on ?

In total, 216 ideas were generated across the four research questions, with 80 unique statements retained after merging duplicates and overlapping concepts (Fig. 2).

### Research question 1: In what circumstances is an inhaler regimen switch appropriate?

**Fig. 3:**
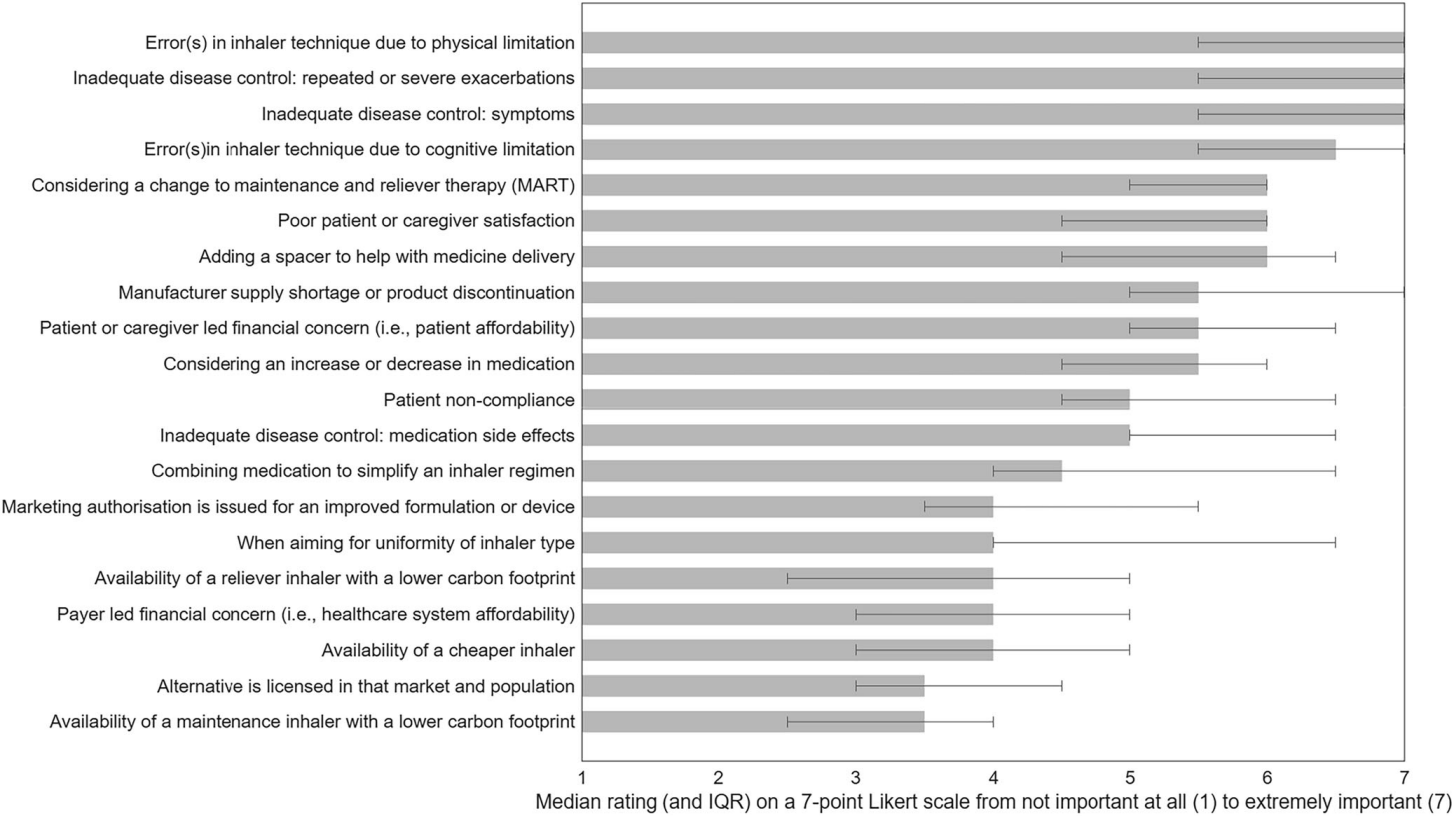
Median importance rating and IQR of each statement generated to question 1: In what circumstance(s) are inhaler regimen switches appropriate? N = 8. Error bars represent IQR of each idea.

The expert panel identified a range of circumstances where an inhaler regimen switch may be appropriate, which covered clinical, patient-related, operational, and environmental factors (Table 2, Fig. 3). Scenarios such as inadequate disease control—whether due to persistent symptoms (median [IQR]: 7 [5.5–7]; 75% ratings ≥6) or repeated/severe exacerbations (7 [5.5–7]; 75%)—were rated most important, alongside errors in inhaler technique caused by physical (7 [5.5–7]; 75%) or cognitive limitations (6–7 [5.5–7]; 75%). Other strongly supported reasons included considering a change to maintenance and reliever therapy (MART) (6 [5–6]; 75%), adding a spacer to improve medicine delivery (6 [4.5–6.5]; 62.5%), and addressing poor patient or caregiver satisfaction (6 [4.5–6]; 62.5%).

**Table 2:** Descriptive and reliability statistics for question 1: In what circumstances are inhaler regimen switches appropriate.

Q1: In what circumstances are inhaler regimen switches appropriate?^†^	Median (IQR)	Ratings ≥ 6, %	Fleiss’ Kappa
Error(s) in inhaler technique due to physical limitation	7 (5.5–7)	75	0.87
Inadequate disease control: repeated or severe exacerbations	7 (5.5–7)	75	0.79
Inadequate disease control: symptoms	7 (5.5–7)	75	0.79
Error(s)in inhaler technique due to cognitive limitation	6–7 (5.5–7)	75	0.93
Considering a change to maintenance and reliever therapy (MART)	6 (5–6)	75	0.83
Poor patient or caregiver satisfaction	6 (4.5–6)	62.5	0.86
Adding a spacer to help with medicine delivery	6 (4.5–6.5)	62.5	0.85
Manufacturer supply shortage or product discontinuation	5–6 (5–7)	50	0.89
Patient or caregiver led financial concern (i.e., patient affordability)	5–6 (5–6.5)	50	0.91
Considering an increase or decrease in medication	5–6 (4.5–6)	50	0.88
Patient non-compliance	5 (4.5–6.5)	37.5	0.89
Inadequate disease control: medication side effects	5 (5–6.5)	37.5	0.86
Combining medication to simplify an inhaler regimen	4–5 (4–6.5)	37.5	0.84
Marketing authorisation is issued for an improved formulation or device	4 (3.5–5.5)	25	0.85
When aiming for uniformity of inhaler type	4 (4–6.5)	37.5	0.80
Availability of a reliever inhaler with a lower carbon footprint	4 (2.5–5)	12.5	0.85
Payer led financial concern (i.e., healthcare system affordability)	4 (3–5)	12.5	0.88
Availability of a cheaper inhaler	4 (3–5)	0	0.85
Alternative is licensed in that market and population	3–4 (3–4.5)	12.5	0.88
Availability of a maintenance inhaler with a lower carbon footprint	3–4 (2.5–4)	0	0.91
*Cronbach’s alpha for question 1:*	0.99		

N = 8.

^†^ Ideas ordered by median rating highest to lowest.

Moderate importance was reported for situations where a switch might be prompted by operational or practical concerns, such as manufacturer supply shortages or product discontinuation (5–6 [5–7]; 50%), patient or caregiver-led financial constraints (5–6 [5–6.5]; 50%), or the need to adjust medication dose (5–6 [4.5–6]; 50%). These factors were considered relevant but less important than clinical need.

In contrast, environmental and cost-related drivers were rated lowest. Availability of inhalers with a lower carbon footprint—whether for maintenance (3–4 [2.5–4]; 0%) or reliever therapy (4 [2.5–5]; 12.5%)—along with payer-led financial concerns (4 [3–5]; 12.5%), availability of an alternative licensed in the market and population (3–4 [3–4.5]; 12.5%), and the availability of cheaper alternatives (4 [3–5]; 0%) were rated of lower importance. Similarly, marketing authorisation for an improved formulation or device (4 [3.5–5.5]; 25%) and efforts to achieve uniformity of inhaler type (4 [4–6.5]; 37.5%) received limited support.

Overall, consensus was strongest for clinical and patient-focused scenarios, with Fleiss’ Kappa values > 0.75 and Cronbach’s alpha >0.70 indicating high internal consistency.

### Research question 2: In what circumstances is an inhaler regimen switch not appropriate?

**Fig. 4:**
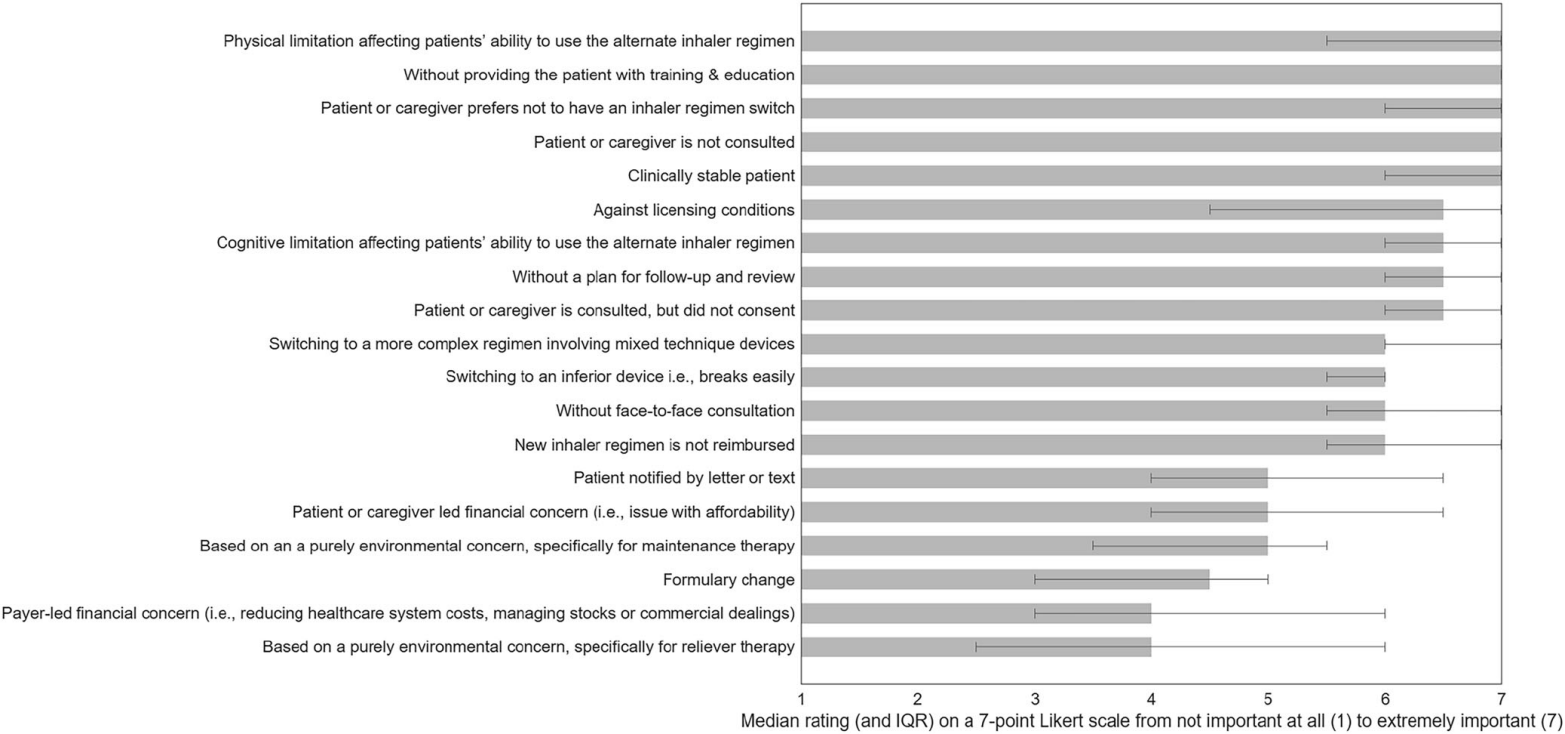
Median importance rating and IQR of each statement generated to question 2: In what circumstance(s) are inhaler regimen switches not appropriate? N = 8. Error bars represent IQR of each idea.

The expert panel identified a broad range of scenarios where inhaler regimen switching is not appropriate, spanning patient-related safeguards, operational factors, regulatory considerations, and environmental or financial drivers (Table 3, Fig. 4).

**Table 3:** Descriptive and reliability statistics for question 2: In what circumstances is an inhaler regimen switch not appropriate?

Q2: In what circumstances is an inhaler regimen switch not appropriate?	Median (IQR)	Ratings ≥ 6, %	Fleiss’ Kappa
Physical limitation affecting patients’ ability to use the alternate inhaler regimen	7 (5.5–7)	75	0.91
Without providing the patient with training & education	7 (7–7)	100	0.97
Patient or caregiver prefers not to have an inhaler regimen switch	7 (6–7)	100	0.97
Patient or caregiver is not consulted	7 (7–7)	100	1.00
Clinically stable patient	7 (6–7)	87.5	0.94
Against licensing conditions	6–7 (4.5–7)	62.5	0.80
Cognitive limitation affecting patients’ ability to use the alternate inhaler regimen	6–7 (6–7)	87.5	0.95
Without a plan for follow-up and review	6–7 (6–7)	100	0.97
Patient or caregiver is consulted, but did not consent	6–7 (6–7)	100	0.97
Switching to a more complex regimen involving mixed technique devices	6 (6–7)	87.5	0.95
Switching to an inferior device i.e., breaks easily	6 (5.5–6)	75	0.92
Without face-to-face consultation	6 (5.5–7)	75	0.91
New inhaler regimen is not reimbursed	6 (5.5–7)	75	0.91
Patient notified by letter or text	5 (4–6.5)	50	0.81
Patient or caregiver led financial concern (i.e., issue with affordability)	5 (4–6.5)	37.5	0.73
Based on an a purely environmental concern, specifically for maintenance therapy	5 (3.5–5.5)	25	0.76
Formulary change	4–5 (3–5)	12.5	0.81
Payer-led financial concern (i.e., reducing healthcare system costs, managing stocks or commercial dealings)	4 (3–6)	37.5	0.83
Based on a purely environmental concern, specifically for reliever therapy	4 (2.5–6)	37.5	0.68
*Cronbach’s alpha for question 2:*	0.97		

N = 8.

^†^ Ideas ordered by median rating highest to lowest.

Patient-related safeguards were rated most important. Switching without patient or caregiver consultation (median [IQR]: 7 [7–7]; 100% ratings ≥6), ignoring patient preference (7 [6–7]; 100%), and proceeding without training and education (7 [7–7]; 100%) or a follow-up plan (6–7 [6–7]; 100%) were unanimously considered inappropriate. Switching clinically stable patients was also highly inappropriate (7 [6–7]; 87.5%). Additional high-priority concerns included introducing more complex regimens involving mixed technique devices (6 [6–7]; 87.5%), switching to inferior devices (6 [5.5–6]; 75%), and cognitive (6–7 [6–7]; 87.5%) or physical limitations (7 [5.5–7]; 75%) that impair inhaler use.

Operational factors were also highlighted. Notifying patients by letter or text was rated as inappropriate (5 [4–6.5]; 50%), as was switching without face-to-face consultation (6 [5.5–7]; 75%) or when the new regimen is not reimbursed (6 [5.5–7]; 75%). Switching against licensing conditions was considered inappropriate (6–7 [4.5–7]; 62.5%).

In contrast, environmental drivers alone—whether for reliever therapy (4 [2.5–6]; 37.5%) or maintenance therapy (5 [3.5–5.5]; 25%)—were rated as weak justifications for switching. Similarly, payer-led financial concerns (4 [3–6]; 37.5%) and formulary changes (4–5 [3–5]; 12.5%) were rated as lower importance, while patient-led affordability concerns were slightly more important but still moderate (5 [4–6.5]; 37.5%).

Overall, agreement was strong for top-rated items (Fleiss’ Kappa >0.80), and Cronbach’s alpha >0.70 indicated high internal consistency.

### Research question 3: What essential activities and timings are required for a successful inhaler regimen switch?

**Fig. 5:**
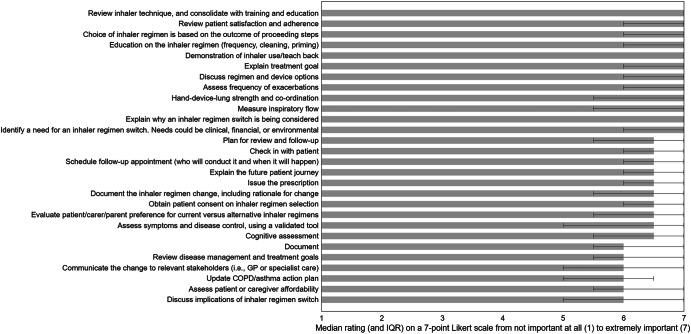
Median importance rating and IQR of each statement generated to question 3: What are the essential activities that need to be taken to implement a successful inhaler regimen switch? N = 8. Error bars represent IQR of each idea.

**Fig. 6:**
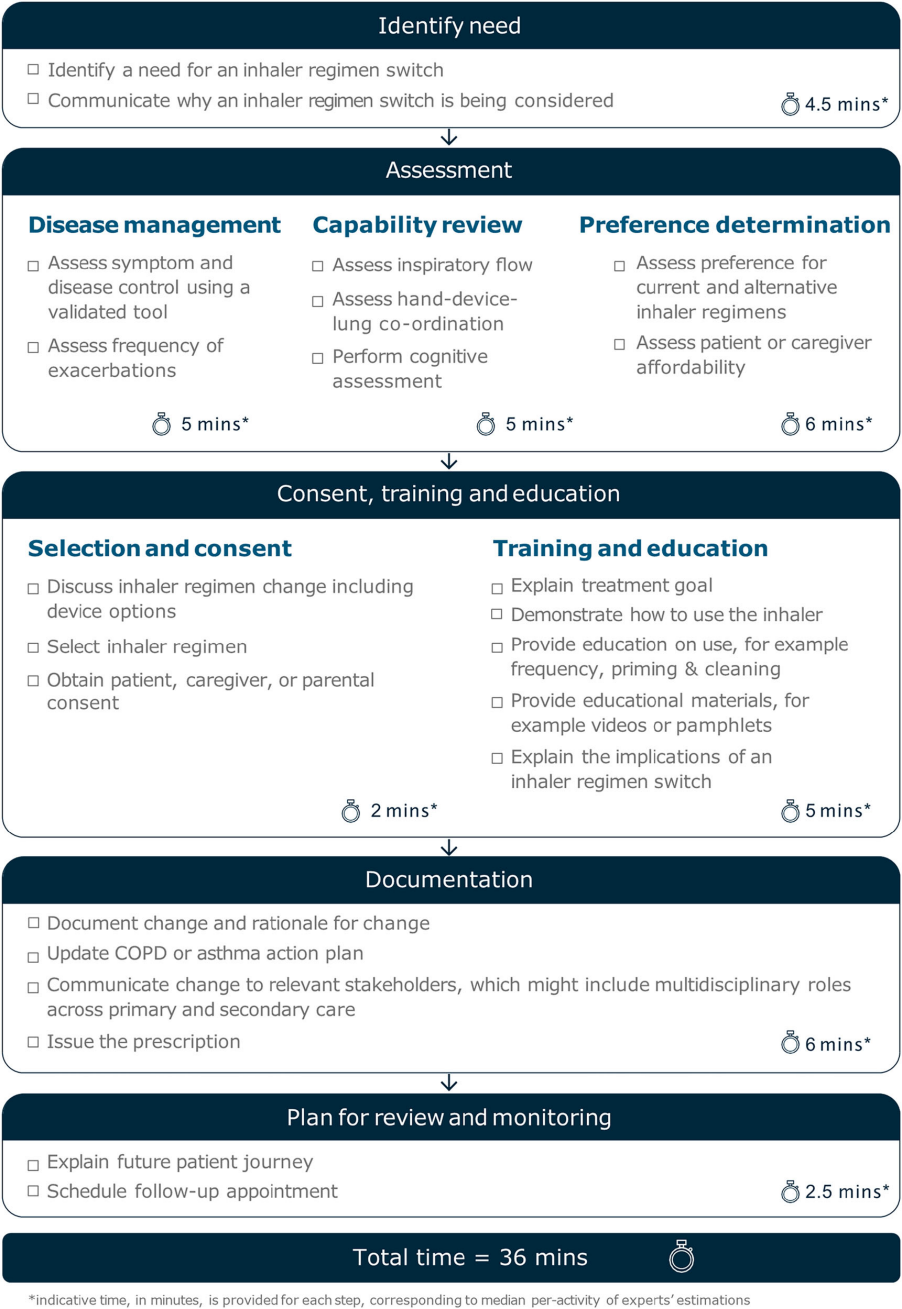
A checklist of essential activities required to implement an inhaler regimen switch, with indicative time required (not including review time). N = *7*.

The expert panel identified a range of activities that they considered essential for effective, safe, and sustainable inhaler regimen switch consultations. Each activity was rated for its overall importance, and assigned an estimated duration in minutes (Table 4, Table 5, Fig. 5). The essential activities were then grouped into a practical checklist, covering identification of need, patient assessment, consent, training & education, documentation and follow-up planning (Fig. 6).

**Table 4:** Descriptive and reliability statistics for question 3: What essential activities [and timings] are required for a successful inhaler regimen switch?

Q3: What essential activities [and timings] are required for a successful inhaler regimen switch?^†^	Median (IQR)	Ratings ≥ 6, %	Fleiss’ Kappa
Review inhaler technique, and consolidate with training and education^‡^	7 (7–7)	100	0.95
Review patient satisfaction and adherence	7 (6–7)	100	0.91
Select inhaler regimen	7 (6–7)	87.5	0.89
Provide education on use (e.g. frequency, cleaning, priming)	7 (6–7)	100	0.97
Demonstrate how to use the inhaler	7 (7–7)	100	0.94
Explain treatment goal	7 (6–7)	100	0.97
Discuss inhaler regimen change including device options	7 (6–7)	87.5	0.93
Assess frequency of exacerbations	7 (6–7)	87.5	0.89
Assess hand-device-lung strength and co-ordination	7 (5.5–7)	75	0.91
Assess inspiratory flow	7 (5.5–7)	75	0.83
Communicate why an inhaler regimen switch is being considered	7 (7–7)	100	0.91
Identify a need for an inhaler regimen switch. (Needs could be clinical, financial, or environmental)	7 (6–7)	100	0.91
Plan for review and follow-up	6–7 (5.5–7)	75	0.97
Check in with patient	6–7 (6–7)	100	0.97
Schedule follow-up appointment (who will conduct it and when it will happen)	6–7 (6–7)	87.5	0.94
Explain future patient journey	6–7 (6–7)	87.5	0.97
Issue the prescription	6–7 (6–7)	87.5	0.95
Communicate why an inhaler regimen switch is being considered	6–7 (5.5–7)	75	0.90
Obtain patient, caregiver, or parental consent	6–7 (6–7)	100	0.92
Assess preference for current and alternative inhaler regimens	6–7 (5.5–7)	75	0.94
Assess symptoms and disease control using a validated tool	6.5 (5–7)	62.5	0.94
Perform cognitive assessment	6.5 (5.5–7)	75	0.95
Document change and rationale for change	6 (5.5–7)	75	0.97
Review disease management and treatment goals	6 (5.5–7)	75	0.97
Communicate the change to relevant stakeholders (i.e., GP or specialist care)	6 (5–7)	62.5	0.95
Update COPD/asthma action plan	6 (5–6.5)	62.5	0.95
Assess patient or caregiver affordability	6 (5.5–7)	75	0.97
Explain the implications of inhaler regimen switch	6 (5–7)	62.5	0.97
*Cronbach’s alpha for question 3:*	0.99		

N = 8

^†^ Ideas ordered by median rating highest to lowest.

^‡^ For example, provide educational materials, for example videos or pamphlets.

**Table 5:** Estimated timings for question 3: What essential activities [and timings] are required for a successful inhaler regimen switch?

Q3: What essential activities [and timings] are required for a successful inhaler regimen switch?^†^	Median time, mins	IQR
Identify need	4.5	3.5–7.5
Disease management	5	3.63–5.75
Capability review	5	5.0–6.88
Preference determination	6	4.25–9.5
Selection and consent	2	1–2.75
Training and education	5	5.0–9.38
Documentation	6	3.5–7.13
Plan for review and monitoring	2.5	1.25–3.75
(Conduct review and monitoring) *excluded from total*	(5)	(5.0–17.5)
**Total by median per-activity time**, min	36	
**Time range (by expert)**, min	23–125	

N = 7.

^†^ Fig. 6 details which ideas are included within the essential activities presented.

All activities identified in question 3 were considered with high importance, with 62.5–100% of ratings being very or extremely important. Activities rated highest in importance with 100% of ratings ≥6 included obtaining patient consent on inhaler regimen selection, explaining treatment goals, demonstrating inhaler use with teach-back, providing education on inhaler regimen (frequency, cleaning, priming), reviewing patient satisfaction and adherence, and consolidating inhaler technique with training. These steps were unanimously considered critical for safe implementation. Other activities considered with high importance encompassed scheduling follow-up appointments, explaining the future patient journey, issuing prescriptions, and discussing regimen and device options. Clinical assessments such as measuring inspiratory flow and evaluating hand-device-lung coordination were also highly rated with ≥75% ratings ≥6. Moderate importance was reported for tasks such as updating COPD/asthma action plans, communicating changes to relevant stakeholders, and reviewing disease management goals, with 62.5–75% ratings ≥6. All activities demonstrated high reliability, with Fleiss’ Kappa values > 0.80 and Cronbach’s alpha >0.70 indicating strong internal consistency.

Time estimates provided by the panel suggest that delivering these activities requires substantial consultation time, with a median of 36 min (range: 23–125) (Fig. 6).

### Research question 4: Who should be involved in decision-making and implementation?

The expert panel identified 13 stakeholder groups involved in decision-making and implementation of inhaler regimen switches and mapped them to six DMU roles: initiator, gatekeeper, buyer, decider, influencer, and user.(Table 6)

**Table 6:**
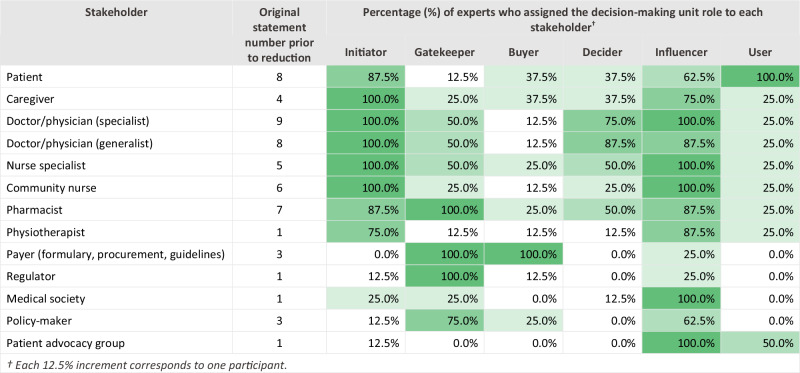
Question 4: In cases where inhaler regimen switches are appropriate, who should be involved in the decision-making and implementation?

Patients and caregivers were central to initiating a switch, with 87.5% of experts assigning the initiator role to patients and 100% to caregivers. Patients were universally recognised as users (100%) and frequently as influencers (62.5%), while caregivers were also seen as strong influencers (75%).

Specialist and generalist physicians, as well as nurse specialists and community nurses, were unanimously considered initiators (100%) and influential in decision-making ( ≥ 87.5%). Physicians were most often assigned the decider role (specialists: 75%; generalists: 87.5%), while nurses were influential but less frequently designated as deciders (50% for nurse specialists; 25% for community nurses). Pharmacists were consistently identified as gatekeepers (100%) and influencers (87.5%), reflecting their role in access and dispensing. While these stakeholders represented six different healthcare professional (HCP) roles, the experts emphasised that HCP roles and responsibilities vary by country and healthcare system. For example, the HCP who has direct contact with a patient with asthma or COPD and is responsible for assessment, review, monitoring, and prescribing will differ across settings. The panel concluded that the influence of each HCP on an inhaler regimen switch depends on skillsets and competencies rather than job titles (Table 7).

**Table 7:** Quality statements on skillsets and competencies required by healthcare professionals influencing and implementing an inhaler regimen switch.

Skillsets and competencies required by healthcare professionals involved in an inhaler regimen switch
Appropriately qualified, with knowledge of respiratory disease management, able to assess disease control and patient capabilities using validated tools, and able to responsibly recommend suitable management strategies.
Understand the inhaler mode of action, handling techniques, and actuation methods, as well as local availability, cost, and reimbursement requirements of different inhaler devices.
Able to deliver appropriate patient training, correct critical errors in technique and direct patients to training support materials (such as videos, leaflets, and infographics).
Able to communicate the need for an inhaler regimen switch and treatment goals to a patient, while being sensitive to their wishes and beliefs.
Be familiar with relevant policy, access, guidelines, and processes relating to inhaler regimen switching.

Payers and regulators were dominant in access control and funding. Both were unanimously assigned gatekeeper roles (100%), with payers also acting as buyers (100%) and occasionally deciders (25%). Regulators were rarely seen as deciders (12.5%) but were acknowledged as gatekeepers for formulary and compliance.

Other stakeholders played supporting roles. Physiotherapists were often considered influencers (87.5%) but rarely deciders (12.5%). Medical societies and patient advocacy groups were strongly associated with influencer roles (100%), while policymakers were viewed as influencers (62.5%) and gatekeepers (75%).

Overall, the panel underscored that decision-making for inhaler regimen switches is not concentrated within a single role but distributed across a diverse set of stakeholders. Initiator roles are primarily held by patients, caregivers, and clinicians, while gatekeeping and purchasing functions rest with payers and regulators. Influencer roles span clinicians, pharmacists, medical societies, and advocacy groups, reinforcing the breadth of perspectives shaping decisions. Patients remain central as both users and key influencers, highlighting the need for shared decision-making. Importantly, the influence of each stakeholder depends more on competencies and context than on formal job titles, reflecting variability across healthcare systems.

### Sensitivity analysis

A jackknife leave-one-out analysis was performed to assess the influence of individual raters on consensus metrics for questions 1, 2, and 3. Across all questions, removal of any single rater did not materially alter median scores or interquartile ranges, indicating stability of central tendency estimates. Fleiss’ Kappa values showed minor fluctuations but generally remained within the same interpretation category (generally >0.80, indicating substantial to almost perfect agreement).

For question 3 timings, Rater 1 exerted the greatest influence on variability; however, overall median consultation time ( ≈ 36 min; range: 33–38.5 min) and IQR remained consistent across iterations. These findings confirm that consensus ratings and estimated time requirements were robust to the exclusion of individual raters, supporting the reliability of the panel’s conclusions.

## Discussion

### Main findings and interpretation of results

This study provides a formalized international expert consensus on when inhaler regimen switches are appropriate, when they should be avoided, and the essential steps and time requirements for safe implementation in asthma and COPD care. Clinical and patient-focused drivers were considered most important for appropriate inhaler regimen switches, which included inadequate disease control, inhaler technique errors, regimen simplification, and supporting patient decisions. Environmental and cost-related drivers were consistently considered of least importance, indicating these should not drive decisions around inhaler regimen switching. Conversely, inappropriate circumstances for switching centred on patient safety. Lack of consultation, consent, education, or follow-up planning were unanimously rated inappropriate, alongside switching clinically stable patients, introducing complex regimens, or ignoring physical/cognitive limitations. These findings underscore the importance of patient involvement and shared decision-making as critical safeguards. Of 28 essential activities assessed, all were considered very to extremely important for an inhaler regimen switch consultation. To operationalise these principles, the panel estimated a required median of 36 min per consultation. Stakeholder mapping revealed that decision-making is distributed across multiple roles: clinicians and caregivers were central as initiators and frequent deciders, while payers and regulators primarily acted as gatekeepers. Patients were universal users and influential participants, reinforcing the need for person-centred, collaborative approaches to inhaler regimen switching.

These findings define clear criteria for appropriate and inappropriate switching and outline practical steps for implementation. They emphasise that inhaler switches should be guided primarily by clinical need and patient involvement rather than cost or sustainability goals, which should be taken into account only once clinical need is covered. The estimated time requirement illustrates the complexity of implementing switches safely and highlights resource implications for healthcare systems.

The research questions, expert discussions, and resulting quality statements were specifically framed around the context of inhaler regimen switching. While principles such as shared decision-making, patient education, and assessment of inhalation technique are broadly applicable and may support inhaler initiation, this study did not directly explore initiation scenarios.

Beyond operational detail, these findings can be situated within behavioural theory. Although this study did not apply a predefined theoretical model, the resulting statements align with the COM-B framework for behaviour change. Successful inhaler regimen switching depends on *Capability* (e.g., patient inhaler technique and HCP training), *Opportunity* (e.g., healthcare system capacity and time for reviews), and *Motivation* (e.g., patient engagement and shared decision-making). Situating the findings within COM-B highlights the multidimensional factors influencing implementation and reinforces the importance of addressing behavioural, structural, and motivational barriers when designing interventions.

### Context and implications

Our results align with previous guidance advocating shared decision-making and patient education as central to inhaler switching (GINA, GOLD, NICE)^31–34^. However, this study expands on prior work by quantifying consensus strength and estimating time requirements, which have not been systematically addressed in earlier frameworks. Unlike policy driven approaches prioritising environmental or cost considerations, this study cautions against these as primary drivers, echoing concerns raised in real-world evidence showing adverse outcomes from non-consented switches^25,35–37^.

Environmental considerations are important and should be taken into account once patient preference and clinical appropriateness have been considered. While inhalers account for <0.03% of global anthropogenic emissions, they represent a notable share of healthcare-related carbon footprint, with pressurised metered-dose inhalers (pMDIs) contributing ~3% within the NHS in England^38^. Some health systems have introduced incentives or regulations to phase out pMDIs based solely on environmental concerns^15,16,39^. These approaches risk compromising patient outcomes if clinical appropriateness is not prioritised. Recent real-world evidence underscores this concern: a Veterans Health Administration formulary change replacing MDIs with DPIs reduced inhaler-related greenhouse gas emissions but was associated with increased HCRU, hospitalisations and pneumonia risk^35^. Importantly, improved disease control is associated with both lower healthcare costs and carbon footprint^26^. Encouragingly, manufacturers are developing next-generation propellants with near-zero or low global warming potential^40–43^. In 2025, the first pMDI medicine containing a next generation propellant was approved by the Medicines and Healthcare products Regulatory Agency in the United Kingdom^42^. This was followed by a positive opinion from the Committee for Medicinal Products for Human Use from the European Medicines Agency, endorsing it for use in the EU^43^. This innovation supports sustainability goals without sacrificing patient care, reinforcing the need for balanced strategies that integrate environmental priorities with clinical and patient-focused decision-making.

Similarly, while cost containment is a legitimate priority for health systems, the expert panel cautioned against switching inhaler regimens solely for payer-led economic reasons. Evidence from previous policy interventions, such as reimbursement restrictions in Iceland, suggests that cost-driven switching without patient consent can lead to poorer disease control, increased short-acting β-agonist use, and higher overall healthcare costs^44^. These unintended consequences reinforce the need for patient-centred and clinically appropriate decision-making rather than purely cost-based policies.

The implications of this expert consensus are significant: they provide a practical framework for clinicians and policymakers to support safe, patient-centred switching and inform resource planning. They also highlight the complexity of implementing switches at scale, given the estimated 36 min consultation time compared with the typical 10 min appointment^45,46^, and the need for adequate resourcing to avoid compromising care quality.

### Strengths and limitations

A key strength of this study is the use of an adapted virtual nominal group technique (vNGT), a structured method for eliciting expert opinion, that is particularly valuable when empirical evidence is limited^47,48^. Its format promotes equitable participation, reduces dominance bias, and prioritises ideas through systematic ranking. Adapting NGT to a virtual setting enabled inclusion of geographically dispersed experts, enhancing feasibility and diversity.

Another strength is the composition of the panel. The multidisciplinary group included respiratory care experts from both primary and secondary care, health economics, and patient advocacy. International representation supports generalisability, although country-specific nuances were not captured.

Limitations include the relatively small group size, which may affect generalisability and allow individual influence^49–51^. However, the sensitivity analysis conducted mitigates this limitation through identifying that excluding one expert in turn does not substantially impact the results. A different panel might have produced different results, and the inclusion of only one patient advocate may limit patient representation despite their extensive advocacy experience.

Lack of anonymisation during discussions could have introduced social desirability bias but impartial facilitation and independent ratings reduced this risk. Finally, industry sponsorship may raise concerns about perceived influence; however, experts were unpaid, and safeguards—independent facilitation and data custody—ensured impartiality and consensus-driven recommendations.

### Future directions

Building on the findings of this expert consensus study, several next steps are recommended to strengthen the applicability and impact of the insights and proposed checklist:

1. Pilot implementation of the checklist in real-world clinical settings to assess feasibility, usability, and integration into routine practice.
2. A Delphi-round confirmation involving a larger and more diverse panel of stakeholders across different healthcare systems to validate and refine the statements.
3. Empirical evaluation of patient outcomes, workflow efficiency, and system-level impact to determine the effectiveness of the checklist in improving inhaler regimen switching.

These steps will help ensure that the framework is both practical and evidence-informed, supporting its translation into clinical and policy contexts.

## Conclusion

The expert panel reported that switching inhaler devices is a complex process that requires significant time, structured planning, and patient engagement. The panel agreed that clinical and patient-focused factors—such as inadequate disease control and inhaler technique errors—should remain the primary drivers of switching decisions, while non-clinical factors, including financial, environmental, and regulatory considerations, should be considered only once clinical and patient-focused aspects have been integrated in the decision process.

To support safe and effective implementation, this study proposes a practical checklist outlining essential activities for initiating and conducting an appropriate switch. Delivering these steps is estimated by the panel to require a median of 36 min per patient, plus time for follow-up reviews—far exceeding the typical 10 min appointment^45,46^. Failure to allocate sufficient time and resources risks undermining care quality and may ultimately be counterproductive, as poorly controlled respiratory disease drives higher costs and HCRU—a major global burden affecting more than 500 million people^1^.

The panel encourages policymakers and healthcare decision-makers to consider the broader impact of safe, sustainable inhaler switching when shaping policy. Prioritising patient-centred, clinically appropriate switching is essential to protect outcomes, optimise resource use, and align with health system goals for quality, efficiency, and sustainability.

## Data Availability

All data generated during this study are included in this published article or its supplementary information files
